# Algorithm-associated digital addiction among older adults: mechanisms and public health implications for healthy aging

**DOI:** 10.3389/fpubh.2025.1746304

**Published:** 2026-01-27

**Authors:** Zhaoxue Li, Xiaoyong Yu, Kan Tian

**Affiliations:** 1 School of Elderly Care Services and Management, Nanjing University of Chinese Medicine, Nanjing, China; 2 Institute of Elderly Care Services and Management, Nanjing University of Chinese Medicine, Nanjing, China; 3 Ruihua Institute of Elderly Care Services and Management, Nanjing University of Chinese Medicine, Nanjing, China

**Keywords:** digital addiction, algorithmic inducement, older adults, personalized recommendation, mobile phone addiction index, healthy aging, digital literacy, China

## Abstract

**Objective:**

To examine how algorithmic inducement mechanisms are associated with digital addiction among Chinese adults aged 60 and above.

**Methods:**

A cross-sectional survey was conducted among older users of major Chinese digital platforms. After data screening, 367 valid questionnaires were analyzed. Digital addiction was measured using the Mobile Phone Addiction Index (MPAI), while a self-developed 16-item scale assessed four algorithmic inducement dimensions: preferential incentives–profit-seeking psychological induction, interactive incentives–emotional compensation induction, stage goals–feedback effect induction, and customized recommendations–exploratory psychological induction. Multiple linear regressions were performed, controlling for age, living arrangement, and daily smartphone use duration.

**Results:**

Digital addiction was widespread, within the sample, with 77.11% of participants scoring in the moderate or higher range. All four algorithmic dimensions were positively associated with digital addiction (*p* < 0.01). Interactive incentives showed the strongest association (*β* = 0.343), followed by preferential incentives (*β* = 0.227); stage goals (*β* = 0.160) and customized recommendations (*β* = 0.163) were smaller yet significant. The model explained 79.1% of the variance (*R*^2^ = 0.791).

**Conclusion:**

Algorithmic inducements—economic, social–emotional, task-feedback, and exploratory mechanisms—are jointly associated with a shift in older adults’ digital use from instrumental participation to immersive dependence. The study introduces an analytical framework, “algorithmic drive–psychological compensation–structural constraint–behavioral reinforcement,” and calls for coordinated algorithm governance and digital literacy initiatives to promote healthy aging in the digital era.

## 1 Introduction

China is experiencing a convergence of population aging and digital transformation, with social aging and informatization becoming increasingly intertwined. Amid both digital dividends and digital divides, the health and social adaptation of older adults has emerged as a pressing public concerns (1, 2). According to the *55th Statistical Report on China’s Internet Development* (2024), the number of Internet users aged 60 and above has reached 199 million, accounting for 64% of the elderly population (3). This finding suggests that, driven by continuous digital inclusion policies and the widespread adoption of information and communication technologies (ICT), the digital divide among older adults is gradually narrowing, creating new opportunities for social participation and personal development. Internet use among this group has increased markedly; their participation in the digital sphere has expanded; and online activities have become an integral part of their social interactions and daily lives (4–7). However, while bridging the digital divide and enhancing online engagement, frequent use of digital platforms may also foster emotional dependence and excessive immersion. Older adults are increasingly showing signs of digital addiction (8–10). Overuse of the Internet and digital platforms can disrupt sleep patterns, foster an “information cocoon” effect through compulsive short-video consumption, and lead to virtual interactions replacing real-world social contact (11–14). Such behaviors not only impair the psychological well-being and social functioning of older adults but also diminish their quality of life and hinder the realization of healthy aging goals. Consequently, digital addiction among older adults has become an emerging public health concern that requires immediate and concerted attention (15). To date, there is no consensus within the academic community on the definition of “digital addiction,” a term that is often used interchangeably with Internet addiction, smartphone addiction, and technology addiction (16). Goldberg (17) first introduced the concept of Internet addiction, defining it as a behavioral disorder in which excessive Internet use interferes with an individual’s daily life, studies, and work. Subsequent research has identified multiple characteristics of digital addiction, including adverse outcomes such as social isolation and neglect of real-world activities, as well as users’ immersive experiences and behavioral dependence (18–22). Some studies have even proposed time-based criteria, defining individuals who use digital devices for more than 4 h per day or over 30 h per week as addicted (23). From an aging research perspective, Du (24) defined digital addiction among older adults as a condition in which prolonged Internet use negatively affects their physical and mental health, as well as their social relationships. This study adopts the same definition. The existing literature has primarily examined the influencing factors of digital addiction among older adults from three dimensions: contextual, individual, and industrial. At the contextual level, the prevalence of digital information flows has reshaped social structures and symbolic environments, subtly transforming older adults’ values and lifestyles (25–27). At the individual level, many older adults have limited digital literacy, making it difficult for them to recognize manipulative design features embedded in digital platforms (28, 29). Driven by loneliness and emotional needs, they may voluntarily rely on digital devices and gradually become trapped in compulsive usage patterns (30, 31). At the industrial level, the rise of the “addictive digital economy” has encouraged enterprises to develop increasingly immersive and manipulative products aimed at capturing the “silver economy” (32–35), while insufficient regulatory oversight has further exacerbated the problem (36).

However, most studies on digital addiction have focused on its psychological factors (37), while overlooking the critical role of digital algorithms that now profoundly shape online behavior. Algorithmic recommendation systems and incentive mechanisms operate through both informational and psychological pathways, serving as important correlates of older adults’ excessive use of digital products (8, 38, 39). Nevertheless, the underlying mechanisms of these algorithms have not been adequately examined, and empirical evidence in this field remains scarce. To address this research gap, the present study constructs a behavioral model of digital addiction among older adults from an algorithmic perspective. Drawing on prior research on digital addiction, this study treats algorithmic inducement mechanisms (as perceived by participants) as the core independent variable. They are categorized into four dimensions: preferential incentives-profit-seeking psychological induction (40–42), interactive incentives-emotional compensation induction (8, 39, 43, 44), stage goals-feedback effect induction (45–47), and customized recommendations-exploratory psychological induction (48–50). The dependent variable is the level of digital addiction, while age, living arrangement, and daily smartphone use duration are included as control variables (51–54). Using a cross-sectional survey and regression analysis, this study empirically examines the associations between algorithmic incentives and digital addiction among older adults. It is hypothesized that all four algorithmic dimensions are positively associated with digital addiction in later life.

## 2 Materials and methods

### 2.1 Participants and sampling

This study focused on Chinese adults aged 60 years and above who used major digital platforms, including Douyin, Kuaishou, Taobao, and Pinduoduo. A combination of convenience and purposive sampling methods was employed to enhance representativeness. The research team distributed questionnaires both online and offline, resulting in 443 responses. Online questionnaires were completed independently via smartphone links, while offline questionnaires were administered with assistance when needed. After excluding invalid samples—such as those with excessively short response times or incorrect answers to attention-check questions—367 valid questionnaires were retained for analysis. Participants were drawn from multiple provinces across China, ensuring regional diversity within the sample. For online participation, informed consent was obtained electronically before questionnaire access; for offline participation, permission was obtained before questionnaire administration. Because recruitment occurred through multiple open channels, a precise response rate could not be calculated. Questionnaires with missing data on key variables were excluded during data screening. Although the sample was collected through a combination of online and offline recruitment, the use of digital platforms may overrepresent older adults who are more digitally engaged or more comfortable using smartphones. This potential sampling bias is acknowledged as a limitation of the study.

### 2.2 Measures

#### 2.2.1 Dependent variable

The dependent variable in this study was the level of digital addiction among older adults. The measurement was based on the Mobile Phone Addiction Index (MPAI) developed by Professor Leung at the University of Hong Kong (55). The MPAI has been widely used in digital addiction research and demonstrates strong psychometric properties. In adapting the Mobile Phone Addiction Index (MPAI) for use with older adults, minor wording refinements were made to improve readability and ensure that all items were easily understood, while preserving the instrument’s original conceptual meaning. The instrument consists of 16 items rated on a five-point Likert scale (1 = never, 5 = always) that assess the degree of compulsive use of digital devices and their adverse effects on daily life. In the present study, the scale demonstrated high internal consistency (Cronbach’s *α* = 0.944). Following Leung (2008), participants ‘addiction scores were classified into four levels based on the total MPAI score (range 16–80): 16–29 (non-addictive), 30–46 (mild addiction), 47–63 (moderate addiction), and 64–80 (severe addiction). Higher scores indicate greater addiction severity. These cut-off values are widely applied in studies involving Chinese adult populations; however, validation studies specifically targeting older adults remain limited, and therefore the thresholds should be interpreted with reasonable caution.

#### 2.2.2 Independent variables

The independent variable in this study was algorithmic inducement behavior, which was assessed using a self-developed questionnaire. The instrument comprised four dimensions: preferential incentives-profit-seeking psychological induction; interactive incentives-emotional compensation induction; stage goals-feedback effect induction; and customized recommendations-exploratory psychological induction. Each dimension included four items, for a total of 16, rated on a five-point Likert scale (1 = strongly disagree, 5 = strongly agree), providing a continuum of responses from negative to positive. Exploratory factor analysis was conducted using principal component analysis with varimax rotation, and factor retention was determined based on eigenvalues, inspection of the scree plot, and theoretical interpretability. The independent variables capture participants’ self-reported perceptions of platform inducement features rather than objective measures of algorithmic exposure.

Item development followed established procedures for scale construction, including literature review, expert consultation, cognitive interviews with older adults, and pilot testing. Wording refinement criteria focused on simplifying sentence structure, removing expressions commonly used by younger users, and ensuring age-appropriate comprehension while preserving the original conceptual meaning of each item. These steps ensured both conceptual clarity and suitability for an older adult population. Example items included: “When I see messages about limited-time discounts or scarce products, I purchase as soon as possible” (preferential incentives); “Browsing likes and comments prompts me to use the application repeatedly” (interactive incentives); “I try to complete platform tasks to avoid missing rewards or points” (stage-goal feedback); and “I continue browsing because I anticipate the next recommended video or post” (customized recommendations). Additional questions, such as “Do you prefer platforms that allow you to choose whether to receive personalized recommendations?” and “Do you think the recommended content helps you obtain useful information or social support?,” were included to collect additional insights for subsequent analyzes.

Reliability and validity analyses demonstrated adequate psychometric properties of the scale. The overall Cronbach’s *α* coefficient was 0.95, with *α* values for the four subscales of 0.851, 0.854, 0.835, and 0.865, respectively. The Kaiser–Meyer–Olkin (KMO) measure was 0.969, and Bartlett’s test of sphericity was significant (*p* < 0.001), indicating the suitability of the data for factor analysis. The analysis supported a four-factor structure, which together explained approximately 71% of the total variance. The retention of four factors was supported not only by statistical criteria but also by theoretical differentiation: economic stimulation, social–emotional feedback, task-based reinforcement, and exploratory recommendation mechanisms represent distinct pathways through which algorithms influence user behavior. To further examine the four-factor structure identified in the exploratory analysis, confirmatory factor analysis was conducted using maximum likelihood estimation to assess model fit. Model fit was evaluated using commonly reported indices, including the comparative fit index (CFI), Tucker–Lewis index (TLI), root mean square error of approximation (RMSEA), and related indicators. The confirmatory analysis showed that all standardized factor loadings exceeded 0.60, supporting the adequacy of the proposed four-factor structure. Both exploratory and confirmatory factor analyses were conducted on the same sample, which may involve a risk of overfitting; therefore, further validation using independent samples or split-sample approaches is warranted in future research.

To enhance methodological transparency, the complete wording of all items—including both the adapted MPAI and the four algorithmic inducement subscales—is provided in Supplementary Appendix 1. Detailed factor loadings, cross-loadings, and item-level reliability statistics are provided in Supplementary Appendix 2.

#### 2.2.3 Control variables

Age, living arrangement, and daily smartphone use duration were included as control variables. Age was controlled because cognitive ability and willingness to adopt new technologies tend to decline with increasing age (56, 57). Living arrangement was included as an indicator of social support, which may influence susceptibility to digital addiction (34, 58). Previous studies have shown that daily smartphone use duration is positively associated with addictive usage patterns (59, 60), highlighting the relationship between smartphone use and the risk of digital addiction.

#### 2.2.4 Conceptual framework

**Figure 1:**
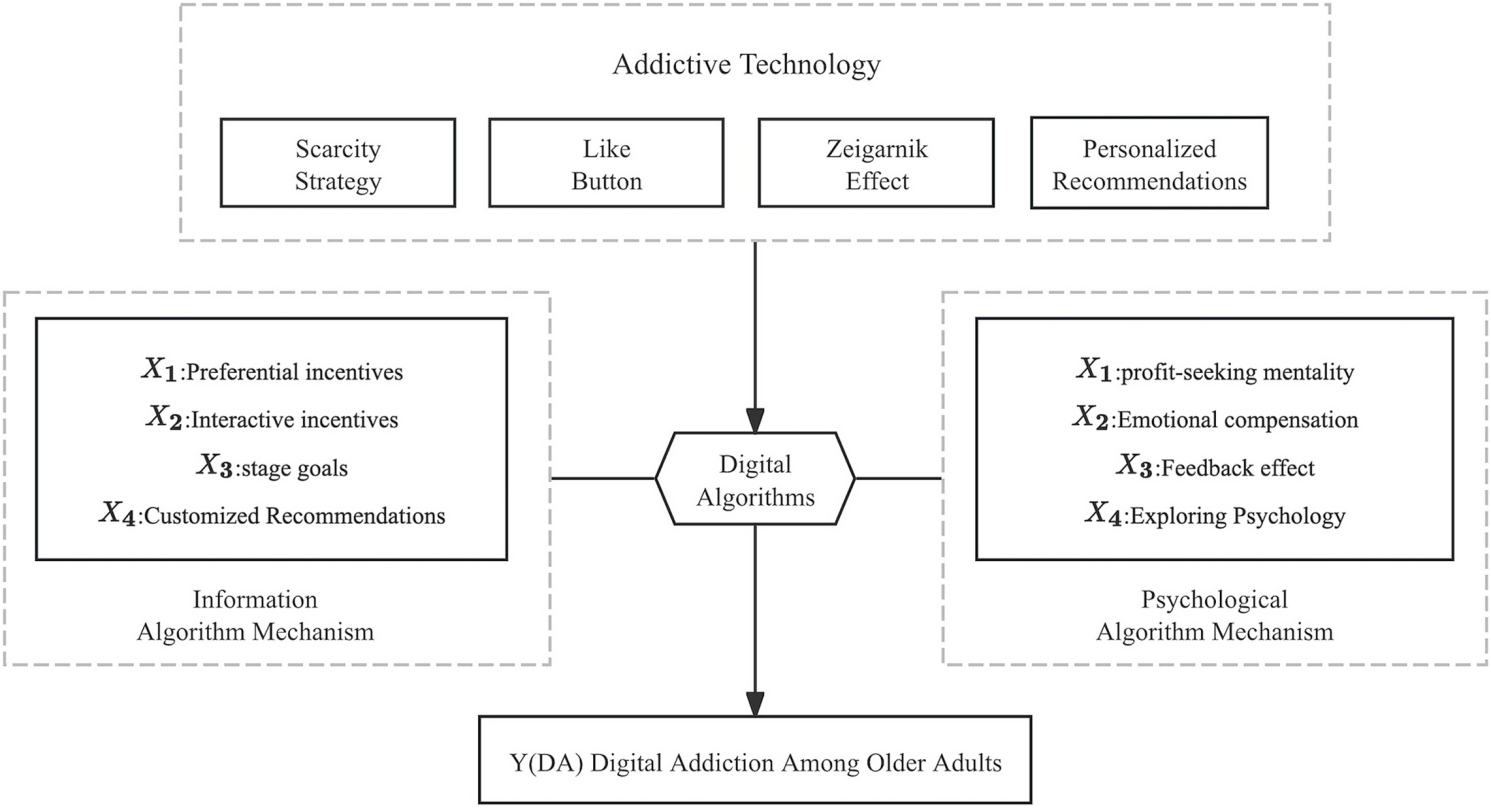
Conceptual model of algorithmic inducement mechanisms and digital addiction among older adults. The model illustrates how algorithmic incentive mechanisms—preferential incentives, interactive incentives, stage-goal feedback, and customized recommendations—are hypothesized to be associated with digital addiction among older adults through informational and psychological pathways.

Based on the theoretical framework and prior literature, this study developed a conceptual model to illustrate how algorithmic inducement mechanisms are hypothesized to be associated with digital addiction among older adults (Figure 1). The model comprises four core dimensions of algorithmic inducement-preferential incentives–profit-seeking psychological induction, interactive incentives–emotional compensation induction, stage goals–feedback effect induction, and customized recommendations–exploratory psychological induction-representing two primary pathways of algorithmic influence: the information algorithm mechanism and the psychological algorithm mechanism. These mechanisms jointly describe how algorithm-enabled mechanisms, through economic, social, and exploratory stimulation, reinforce habitual engagement and may be linked to digital addiction.

### 2.3 Statistical analysis

All statistical analyzes were performed using IBM SPSS Statistics version 29. Descriptive statistics were first conducted to present the demographic characteristics of the sample. Subsequently, multiple linear regression analyzes were performed, with the level of digital addiction as the dependent variable, the four dimensions of algorithmic inducement as independent variables, and age, living arrangement, and daily smartphone use duration as control variables. Age was entered as a continuous variable. Living arrangement and daily smartphone use duration were entered as numeric variables reflecting ordered categories as recorded in the dataset and were treated as ordinal predictors, assuming a monotonic relationship with digital addiction severity. A significance level of *p* < 0.05 was used. The variance inflation factor (VIF) was calculated to assess multicollinearity, and all values were below 5, indicating no severe multicollinearity.

### 2.4 Ethics statement

Approval for this study was exempted by the Ethics Committee of Nanjing University of Chinese Medicine, as the research involved an anonymous, non-interventional questionnaire survey and collected no identifiable personal information. The study meets the requirements of the National Statement on Ethical Conduct in Human Research (2007) and adheres to the principles of the Declaration of Helsinki.

Before accessing the questionnaire, all participants were informed of the study purpose, the voluntary nature of participation, the anonymity of responses, and their right to withdraw at any time. Completion of the survey was considered to indicate implied informed consent.

Given that the participants were older adults, several additional safeguards were implemented, including simplified item wording, reduced cognitive burden, and the option to skip any questions that caused discomfort. No physical, psychological, social, or economic risks were imposed on participants.

## 3 Results

### 3.1 Sample characteristics

A total of 367 valid samples were obtained in this study, covering participants from multiple provinces across China, thereby ensuring a certain degree of representativeness. Table 1 presents the basic demographic characteristics of the sample. The majority of respondents were aged between 60 and 70 years (85.84%), representing the primary group currently engaged in digital participation. Regarding Internet use, 74.65% of older adults reported using the Internet for more than 2 h per day. Data on commonly used applications indicated that Douyin and WeChat were the most frequently used platforms, with 88.56% of older adults regularly using them, underscoring their prominent role in their digital lives.

**Table 1:** Demographic and internet use characteristics of the sample (*n* = 367).

Variable	Category	*n* = 367	Percentage (%)
Age (years)	60–65	119	32.43
	66–70	196	53.41
	71–75	27	7.36
	76–80	14	3.81
	≥80	11	3.00
Living arrangement	Living alone	77	20.98
	Living with spouse	163	44.41
	Living with children	80	21.81
	Living in nursing home	45	12.26
	Other	2	0.54
Daily Internet use (hours)	<1	40	10.90
	1–2	53	14.44
	2–3	110	29.97
	3–5	102	27.79
	>5	62	16.89
Commonly used applications	WeChat	238	64.85
	Douyin (TikTok China)	325	88.56
	Taobao	48	13.08
	Pinduoduo	124	33.79
	Others	14	3.81

This table presents the participants’ age distribution, living arrangements, daily internetuse duration, and commonly used applications among older adults across multiple provinces in China.

Table 1 summarizes the demographic and Internet use characteristics of the participants, including their age distribution, living arrangements, daily Internet use, and commonly used applications.

### 3.2 Levels of digital addiction among older adult

Based on the measurement results of the Mobile Phone Addiction Index (MPAI), significant variations in digital addiction levels were observed among older adults (Table 2). The mean MPAI score in the sample was 54.20 (SD = 14.18), indicating a generally elevated but heterogeneous level of digital addiction among older adults. Only 13.35% of participants showed no signs of digital addiction, while 9.54% exhibited mild addiction. The proportion of moderate addiction was the highest at 49.32%, followed by severe addiction at 27.79%. Overall, more than three-quarters of the respondents were at moderate or higher levels of digital addiction, indicating that digital addiction is relatively prevalent among the older adult population.

**Table 2:** Distribution of digital addiction levels among older adults.

Addiction Level	*n* = 367	Percentage (%)	Score range
Non-addictive	49	13.35%	16–29 points
Mild addiction	35	9.54%	30–46 points
Moderate addiction	181	49.32%	47–63 points
Severe addiction	102	27.79%	64–80 points

Digital addiction levels were assessed using the Mobile Phone Addiction Index (MPAI), categorized into four levels: Non-addictive, mild, moderate, and severe. Higher scores indicate greater addiction severity.

### 3.3 Regression analysis results

To examine the relationship between digital addiction and algorithmic inducement behaviors among older adults, two multiple linear regression models were constructed (Tables 3 and 4). Model 1 included only the control variables, while Model 2 incorporated the four core independent variables along with the control variables. Importantly, all regression analyses treated MPAI as a continuous outcome, and the observed associations remained robust when digital addiction was operationalized using continuous scores rather than categorical cutoffs.

**Table 3:** Regression results for control variables (dependent variable = digital addiction).

Variable	*β*	*t*	*p*	VIF
Age	0.023	0.525	0.600	1.008
Living arrangements	−0.164	−3.695	<0.001	1.025
Daily use duration	0.502	11.319	<0.001	1.028

Model 1 includes only the control variables (age, living arrangement, and daily use duration). Coefficients (*β*), *t*-values, and significance levels (*p*-values) are reported.

**Table 4:** Regression results including control and independent variables (dependent variable = digital addiction).

Variable	*β*	*t*	*p*	VIF
Age	0.016	0.656	0.512	1.011
Living arrangements	−0.006	−0.254	0.800	1.084
Daily use duration	0.131	4.745	<0.001	1.318
Preferential incentives—profit-seeking psychological induction	0.227	5.273	<0.001	3.194
Interactive incentives—emotional compensation induction	0.343	7.578	<0.001	3.511
Stage goals—feedback effect induction	0.160	3.200	0.001	4.297
Customized recommendations—exploratory psychological induction	0.163	3.850	<0.001	3.080

Model 2 incorporates the four algorithmic inducement dimensions-preferential incentives, interactive incentives, stage-goal feedback, and customized recommendations-alongside the control variables. All independent variables are positively and significantly associated with digital addiction among older adults.

#### 3.3.1 Results for control variables

In Model 1, the effect of age on the level of digital addiction was not significant (*β* = 0.023, *p* = 0.600), indicating that differences across age groups were insufficient to produce a significant effect. Living arrangement showed a significant negative correlation with digital addiction (*β* = −0.164, *p* < 0.001), suggesting that certain factors related to living conditions may reduce the degree of digital addiction among older adults. Daily smartphone use duration was significantly and positively associated with digital addiction (*β* = 0.502, *p* < 0.001), implying that longer periods of online engagement correspond to higher levels of digital addiction.

In Model 2, age and living arrangement were no longer significant (*p* > 0.05), indicating that their effects were partially absorbed by the core independent variables. The effect of daily smartphone use duration decreased (*β* = 0.131, *p* < 0.001) but remained significant, indicating that this variable maintained stable explanatory power for digital addiction.

#### 3.3.2 Results for independent variables

In Model 2, all four dimensions of algorithmic inducement behavior were significantly and positively associated with digital addiction among older adults. Preferential incentives-profit-seeking psychological induction (*β* = 0.227, *p* < 0.001) showed a significant positive relationship with the level of addiction, while interactive incentives-emotional compensation induction exhibited the strongest effect (*β* = 0.343, *p* < 0.001), indicating that interactive feedback is a key factor explaining variations in digital addiction. Stage goals-feedback effect induction (*β* = 0.160, *p* = 0.001) and customized recommendations-exploratory psychological induction (*β* = 0.163, *p* < 0.001) also showed significant positive associations with addiction levels. Comparison of standardized coefficients revealed that interactive incentives-emotional compensation induction had the strongest influence, followed by preferential incentives, while the effects of stage goals and customized recommendations were smaller but still significant.

#### 3.3.3 Model fit and significance

Collinearity diagnostics indicated that all variance inflation factor (VIF) values were below 5, suggesting that the model did not suffer from serious multicollinearity. Regression diagnostics were conducted by inspecting residual normality (P–P plots), residual-versus-fitted plots for heteroscedasticity, and influence statistics (Cook’s distance and leverage). The diagnostic outputs are provided in the Supplementary materials. The coefficient of determination (*R*^2^) for Model 2 was 0.791, indicating that the control and independent variables together explained 79.1% of the variance in the dependent variable. Analysis of variance further demonstrated that the overall model was significant (*F* test, *p* < 0.05), confirming that the regression equation exhibited a good model fit.

## 4 Discussion

This study conducted a comprehensive analysis of the formation mechanism of digital addiction among Chinese adults aged 60 years and above, focusing on algorithmic inducement behaviors. The results, derived from a large sample size, revealed that digital addiction among older adults is not a sporadic issue but a widespread and structural one, with 77.11% of participants falling into the moderate or higher levels of addiction category. This finding should be interpreted with caution. Although the MPAI cutoffs proposed by Leung (2008) are widely used, validation studies specifically targeting older adult populations remain limited. In addition, given the sampling characteristics described above—particularly the reliance on convenience and purposive recruitment, and the high prevalence of intensive use of short-video platforms—the observed distribution may not reflect prevalence estimates in the broader older adult population. Nevertheless, this pattern suggests that digital immersion has become a salient phenomenon within this sample of older adults. More broadly, as Internet use becomes more prevalent, older adults are not only catching up in terms of technology adoption but also becoming increasingly dependent within an algorithm-mediated digital environment (61). The study found that all four dimensions of algorithmic inducement-preferential incentives, profit-seeking psychological induction, interactive incentives, emotional compensation induction, stage goals, feedback effect induction, and customized recommendations, exploratory psychological induction-were significantly and positively associated with digital addiction. Among these, interactive incentives–emotional compensation induction had the strongest effect, highlighting the critical influence of social feedback mechanisms in promoting media immersion among older adults (62). Preferential incentives–profit-seeking psychological induction ranked second, indicating the continued importance of economic stimulation in influencing usage behaviors in this group (63). Although stage goals–feedback effect induction and customized recommendations–exploratory psychological induction showed relatively weaker effects, both remained statistically significant. The model’s coefficient of determination (*R*^2^ = 0.791) indicated that the four dimensions, together with the control variables, explained 79.1% of the variance in digital addiction, demonstrating a good overall model fit. Although the model accounts for a substantial proportion of variance (*R*^2^ = 0.791), the findings should be interpreted with appropriate caution. Because all variables were measured using self-report instruments at a single time point, some degree of shared-method variance cannot be entirely ruled out, and the cross-sectional design does not permit firm causal conclusions. A Harman single-factor test indicated that the first unrotated factor accounted for 52.51% of the variance, suggesting that common-method variance may be present. Nevertheless, the consistency of the results across multiple algorithmic dimensions provides convergent support for the robustness of the associations observed.

The findings indicate that digital addiction among older adults is not merely a psychological phenomenon but rather the result of multiple structural factors acting in combination, specifically reflecting the interplay of individual, technological, and institutional dilemmas (64–66). Older adults, constrained by cognitive decline and weakened social support, are more likely to develop psychological dependence within algorithm-mediated digital environments (64, 67, 68). Meanwhile, algorithms operate under the core logic of “attention maximization” (69), reinforcing usage behaviors through reward feedback and personalized recommendations (70–72). Immediate reward engagement and time preferences play significant roles in compulsive use behaviors. Hyperbolic discounting accounts for this phenomenon by positing that individuals tend to prioritize short-term rewards over long-term gains (73). While existing research suggests a correlation between these mechanisms and addictive behaviors (74, 75), it remains to be further validated whether they directly lead to compulsive use. Digital platforms, particularly recommendation systems, exploit this by offering continuous feedback, triggering immediate gratification, and reinforcing repetitive behaviors. Manipulative platform designs further intensify this by enhancing user engagement through constant feedback mechanisms, solidifying the cycle of addiction (76). This process involves reward learning and cue reactivity, where users become increasingly dependent on instant rewards, leading to habitual and compulsive use (77). At the institutional level, the existing digital governance system remains insufficient in terms of algorithmic ethics and user protection. Weak regulatory mechanisms and low algorithmic transparency expose older adults to risks of commercial manipulation and content control (78, 79).

Specific mechanisms, such as preferential incentives–profit-seeking psychological induction, showed a significant positive association with digital addiction among older adults (*B* = 0.190, *p* = 0.001). The data revealed that 54.23% of respondents reported that they “purchase as soon as possible when seeing notifications of limited-time offers or product scarcity,” and 59.13% believed that “flash sales or purchase restrictions make them shop more frequently.” These findings highlight the significant influence of economic incentives on the behavior of older adults. Digital platforms, through coupons, limited-time discounts, and point-based reward tasks, effectively create a sense of “scarcity,” triggering immediate behavioral responses (80–82) and reflecting the sophisticated strategies employed in digital marketing. At the psychological level, older adults have long been influenced by frugality-oriented values such as “thriftiness” and “saving whenever possible.” When facing algorithmically constructed situations of virtual scarcity, they are more likely to experience “opportunity anxiety,” leading to impulsive purchasing and repetitive use behaviors (83–85). The “scarcity effect” (86, 87) and “loss aversion theory” (88) in behavioral economics provide a sound theoretical explanation for this phenomenon. As algorithmic mechanisms continuously amplify these economic stimuli, rational saving behavior gradually transforms into irrational immersion, forming an economically driven pathway to digital addiction and illustrating the potential risks associated with this phenomenon.

Interactive incentives–emotional compensation induction, referring to the emotional rewards or gratifications that individuals receive from digital interactions, had the most pronounced effect (*B* = 0.293, *p* = 0.001), serving as a core variable most strongly associated with digital addiction among older adults. The survey results indicated that 59.13% of respondents agreed that “likes, comments, and other feedback encourage repeated use of the application,” while 59.67% reported that “receiving replies from others or gaining followers brings a sense of pleasure.” These findings suggest that algorithm-enabled platform features may reinforce older adults’ media engagement through emotional incentive mechanisms. Due to the weakening of social roles and limited real-world interactions, older adults are more inclined to seek emotional feedback and a sense of social presence through online communication (89–91). Features embedded in algorithms, such as likes, comments, and follower notifications, provide immediate feedback and psychological gratification, partially compensating for diminished social roles (92, 93). However, the immediacy and selectivity of such feedback may make older adults more susceptible to virtual dependence, which may gradually evolve into emotionally driven digital addiction (55).

Third, stage goals–feedback effect induction was also significantly and positively associated with digital addiction among older adults (*B* = 0.142, *p* = 0.001). A total of 61.04% of respondents indicated that they “try to complete platform tasks to avoid the expiration of rewards or coins,” while 62.94% reported that they “often or always continue watching short videos to learn about subsequent content.” This mechanism can be explained by the Zeigarnik effect in psychology, which posits that unfinished tasks create psychological tension and a persistent drive, motivating individuals to repeat behaviors until a sense of completion and psychological equilibrium is achieved (94). Digital platforms transform this tension into motivation for continued engagement through mechanisms such as point rewards, task unlocking, and level systems, leading older adults into a behavioral cycle of “unfinished tasks–repeated logins–reinforced feedback” (95–97). This task-driven mechanism substantially increases usage frequency and, over time, strengthens media dependence and habitual behavioral patterns (98).

Finally, customized recommendations–exploratory psychological induction also showed a significant effect (*B* = 0.149, *p* = 0.001). A total of 64.31% of older adults reported that they “continue browsing because they anticipate the next video,” and 65.67% agreed that “recommended content aligns with their personal interests and needs.” Algorithmic recommendation systems, driven by big data analytics and user profiling, continuously optimize their delivery models to achieve precise content matching (99, 100). While this mechanism reduces information search costs, it simultaneously narrows the diversity of information sources (101). Curiosity and the desire for recognition further drive older adults to become immersed in content environments shaped by algorithmic recommendations (64, 102). As recommendation systems self-optimize, platforms establish a closed loop of “interest modeling–content delivery–behavioral feedback–algorithmic reinforcement,” gradually leading users to develop personalized dependence (99). The dual nature of customized recommendations lies in their ability to enhance user experience while simultaneously reinforcing the “information cocoon effect,” fostering a continuous exploratory form of addiction at the psychological level (13, 103). Overall, algorithmic inducement behaviors, through four psychological pathways-economic stimulation, which involves the anticipation of rewards; emotional compensation, which provides a sense of comfort or satisfaction; goal-driven feedback, which reinforces the achievement of specific objectives; and exploratory reinforcement, which encourages the discovery of new content-are jointly associated with a shift in older adults’ digital usage from instrumental participation to emotional dependence (104). The intertwining of psychological compensation and technological reinforcement constitutes the core mechanism underlying the formation of digital addiction among older adults.

Furthermore, this study provides important implications at both theoretical and practical levels. Theoretically, it proposes an analytical framework of “algorithmic drive–psychological compensation–structural constraint–behavioral reinforcement,” revealing the underlying logic of digital addiction. This framework is intended as an interpretive lens for organizing the observed associations rather than a causal model. Digital addiction among older adults is not merely a technological outcome but the result of interactions among psychological needs, algorithmic mechanisms, and social structures. This finding expands the generational perspective of digital addiction research and offers a new theoretical foundation for understanding the psychological vulnerability and media adaptation mechanisms of older adults in an algorithmic society. In particular, understanding the interplay between algorithmic designs and older adults’ psychological traits is critical in addressing algorithmic exploitation and addiction risks (105). Older adults, often less equipped to recognize persuasive digital cues, face heightened risks of algorithmic manipulation, which may exacerbate their vulnerability to digital addiction (7, 39). In practice, the findings have significant implications for governments, platforms, and social governance. Governments should strengthen regulatory frameworks for algorithmic ethics and user protection to prevent commercial platforms from exploiting addictive design features that may harm vulnerable groups (66, 106). More specifically, regulatory agencies could develop age-sensitive guidelines limiting the intensity and frequency of reward-based inducements, require clearer disclosures about how recommendation algorithms prioritize content, and establish auditing mechanisms to evaluate potential risks to older adults. Platforms should improve algorithmic transparency, reduce reward-based inducements and excessive personalized recommendations, and implement reminders that promote healthy usage habits (107, 108). For example, platforms could offer optional low-stimulation modes for older adults, provide gentle prompts during prolonged screen time, and design simplified controls allowing users to adjust or disable personalized recommendations. Communities and families should enhance digital literacy education and provide psychological support to help older adults identify algorithmic inducement behaviors, fostering rational internet use and balanced social engagement (109, 110). Concrete literacy training could include teaching older adults how to recognize persuasive cues (such as scarcity prompts or emotional feedback), manage notifications, and set personal usage boundaries. In conclusion, coordinated governance across multiple sectors can achieve a balance between technological advancement and social protection, thereby fostering an inclusive and safe digital ecosystem that promotes active aging and advances the goal of digital well-being.

## 5 Conclusion

This study examined the formation mechanism of digital addiction among Chinese adults aged 60 years and above from the perspective of algorithmic inducement behaviors. The findings revealed a dual pattern in the digitalization process of older adults-bridging the digital divide while falling into digital dependency. Platform design features and algorithm-enabled mechanisms, through mechanisms such as economic incentives, social feedback, task incentives, and content recommendations, may fulfill the psychological compensation needs of older adults while simultaneously reinforcing habitual use and emotional attachment. These mechanisms collectively may help explain the transition from instrumental to immersive digital engagement. Overall, the study uncovers the generative logic of digital addiction among older adults: the external inducement of algorithmic drivers interacts with individual psychological compensation needs, forming a cyclical mechanism of behavioral dependence and immersive use under the structural constraints of technological logic and institutional environments. These findings suggest that digital addiction among older adults is not merely a psychological or technological issue but rather the outcome of the combined effects of algorithmic environments, social support, and governance mechanisms. Theoretically, this study extends the generational perspective in digital addiction research and proposes an explanatory framework- “algorithmic drive–psychological compensation–structural constraint–behavioral reinforcement.” This framework provides a new analytical lens for understanding media adaptation and psychological vulnerability among older adults in algorithmic societies. In practice, the study emphasizes the importance of collaboration between digital governance and social support. A balance should be sought between technological innovation and social protection to prevent algorithmic inducements from intensifying digital dependence among older adults and to promote the development of an inclusive, safe, and sustainable digital ecosystem for the aging population. Despite revealing the association between algorithmic mechanisms and digital addiction through survey data, this study has certain limitations. First, although the study identifies strong associations between algorithmic inducement and digital addiction, its cross-sectional design does not allow for firm causal inferences. Second, although both online and offline recruitment were used, the sampling strategy may still overrepresent older adults who are more digitally active or more familiar with smartphone-based surveys, thereby limiting representation of less-connected groups. Third, all variables relied on self-report measures rather than behavioral log data; thus, recall bias and shared-method variance cannot be entirely ruled out. Fourth, the study was conducted within the sociocultural and platform ecosystem of mainland China, and the dynamics of algorithmic inducement may differ in other cultural or regulatory contexts. Future research could employ longitudinal tracking and behavioral log data to further explore the dynamic relationships between algorithmic feedback and psychological dependence. It should also focus on the long-term impacts of digital environments on older adults’ cognition, social interactions, and quality of life, thereby providing stronger empirical support for healthy aging and digital governance in an increasingly algorithmic society.

## Data Availability

The raw data supporting the conclusions of this article will be made available by the authors, without undue reservation.
