# Supplementary material for: Algorithm-associated digital addiction among older adults: mechanisms and public health implications for healthy aging
Source: Front Public Health. 2026 Jan 27;13:1746304. doi: 10.3389/fpubh.2025.1746304 (PMC12885990; doi:10.3389/fpubh.2025.1746304)
Supplement: Supplementary file 1 [file Data_Sheet_1.PDF]

Supplementary Appendix 1:

Digital Addiction and Algorithmic Inducement Questionnaire (English Version)

This appendix provides the full English wording of all questionnaire items used in the study, including the adapted Mobile Phone Addiction Index (MPAI) and the self-developed Algorithmic Inducement Scale.

Section A. Basic Information

1. Gender:

□ Male

□ Female

2. Age: ______

3. Place of residence:

□ Urban

□ Rural

4. Living arrangement:

□ Living alone

□ Living with spouse

□ Living with children

□ Living in an elderly care institution

5. Education level:

□ Primary school or below

□ Middle school

□ High school

□ Junior college

□ Undergraduate

□ Master’s degree or above

6. Daily internet use time:

□ Less than 1 hour

□ 1–2 hours

□ 2–3 hours

□ 3–5 hours

□ More than 5 hours

7. Frequently used applications (multiple choice):

□ WeChat

□ Douyin (TikTok)

□ Taobao

□ Pinduoduo

□ Others: ______

Section B. Digital Addiction (Adapted MPAI)

Scoring: 1 = strongly disagree, 5 = strongly agree

1. My friends or family have complained about my use of digital devices.

2. Someone has told me that I spend too much time on digital devices.

3. I have tried to hide how much time I spend on digital devices.

4. I find myself using digital devices longer than I originally intended.

5. I try to reduce my time on digital devices but cannot succeed.

6. I never feel that I have spent enough time on digital devices.

7. When there is no internet, I worry about missing out on information.

8. I find it difficult to turn off digital devices.

9. I become anxious if I do not check my device for a while.

10. I feel restless without digital devices.

11. I spend more time online and less time interacting with people around me.

12. My family interactions have decreased because of my online use.

13. My leisure activities have decreased because of my online use.

14. My mood worsens if I do not go online for a period of time.

15. I become so absorbed in digital devices that I neglect other necessary tasks, causing problems.

16. Spending time on digital devices reduces my work efficiency.

Section C. Algorithmic Inducement Behaviors

Scoring: 1 = strongly disagree, 5 = strongly agree

Preferential Incentives — Profit-Seeking Psychological Inducement

1. I sign in daily to receive coupons or cash rewards provided by the platform.

2. When I see notifications about limited-time offers or scarce products, I purchase immediately.

3. Limited-time promotions or discounts make me shop more frequently.

4. I prefer using applications that allow me to gain monetary benefits.

Interactive Incentives — Emotional Compensation Inducement

5. Browsing likes, comments, and other feedback makes me repeatedly use the application.

6. I feel needed or recognized when interacting with others through digital platforms.

7. I feel happy or satisfied when others reply to me or when my follower count increases.

8. Compared to offline interactions, I prefer sharing my thoughts online.

Stage Goals — Feedback Effect Inducement

9. Virtual games on platforms (such as virtual farming or task-based activities) attract me to log in continuously.

10. I try to complete platform tasks to avoid the expiration of rewards or coins.

11. Unfinished short-video or livestream content often makes me continue watching.

12. I extend my device usage time to obtain platform rewards.

Customized Recommendations — Exploratory Psychological Inducement

13. I continue browsing because I anticipate the next recommended video or content.

14. The content pushed to me usually matches my interests and needs.

15. Recommended content makes me feel understood and suits my current situation.

16. Personalized recommendations make me interested in topics I previously ignored.

Section D. Attitudes Toward Algorithmic Recommendations

1. Do you carefully read the privacy policy of applications?

☐ Never

☐ Sometimes

☐ Often

2. Would you choose a platform that helps reduce addictive usage?

☐ Unwilling

☐ Not sure

☐ Very willing

3. Do you support legislation or technical restrictions that limit certain content recommendations for older adults (e.g., anti-addiction mechanisms for short videos)?

☐ Strongly oppose

☐ Oppose

☐ Not sure

☐ Support

☐ Strongly support

4. Would you like platforms to provide options that allow you to control whether to receive recommended content?

Scoring: 1 = completely unwilling, 5 = very willing

5. Do you think recommended content helps you obtain useful information or social support?

Scoring: 1 = strongly disagree, 5 = strongly agree
