# Supplementary material for: Algorithm-associated digital addiction among older adults: mechanisms and public health implications for healthy aging
Source: Front Public Health. 2026 Jan 27;13:1746304. doi: 10.3389/fpubh.2025.1746304 (PMC12885990; doi:10.3389/fpubh.2025.1746304)
Supplement: Supplementary file 2 [file Data_Sheet_2.docx]

Supplementary Appendix 2

This appendix provides supplementary psychometric analyses and regression diagnostics supporting the main analyses reported in the manuscript.

**Table S1. Exploratory Factor Analysis (EFA) Results for the Algorithmic Inducement Scale**

Rotated factor loadings based on principal component analysis with varimax rotation.

| Item | Factor 1  (Preferential incentives) | Factor 2  (Interactive incentives) | Factor 3  (Stage goals) | Factor 4  (Customized recommendations) |
| --- | --- | --- | --- | --- |
| A1 |  |  | **0.683** |  |
| A2 |  |  |  | **0.774** |
| A3 |  |  |  | **0.754** |
| A4 |  |  | **0.774** |  |
| B1 |  | **0.534** | 0.473 |  |
| B2 | 0.444 | **0.679** |  |  |
| B3 |  | **0.681** |  |  |
| B4 |  | **0.747** |  |  |
| C1 |  | **0.571** |  | 0.408 |
| C2 |  | 0.445 | **0.483** |  |
| C3 | **0.678** |  |  |  |
| C4 |  | 0.483 | **0.565** |  |
| D1 | **0.684** |  |  |  |
| D2 | **0.729** |  |  |  |
| D3 | **0.742** |  |  |  |
| D4 | **0.666** |  |  |  |

**Note.** Factor loadings ≥ 0.40 are reported. Primary loadings are highlighted in bold.

Rotation converged in 7 iterations.

**Table S2. Item-level Reliability Statistics for the Algorithmic Inducement Scale (N = 367)**

| Item | Corrected Item–Total Correlation | Cronbach’s α if Item Deleted |
| --- | --- | --- |
| A1 | 0.726 | 0.947 |
| A2 | 0.717 | 0.947 |
| A3 | 0.717 | 0.947 |
| A4 | 0.684 | 0.948 |
| B1 | 0.729 | 0.947 |
| B2 | 0.726 | 0.947 |
| B3 | 0.722 | 0.947 |
| B4 | 0.707 | 0.947 |
| C1 | 0.675 | 0.948 |
| C2 | 0.733 | 0.947 |
| C3 | 0.702 | 0.947 |
| C4 | 0.783 | 0.946 |
| D1 | 0.736 | 0.947 |
| D2 | 0.697 | 0.947 |
| D3 | 0.722 | 0.947 |
| D4 | 0.719 | 0.947 |

Note. All corrected item–total correlations exceeded 0.60, and deletion of any single item did not improve the overall internal consistency (Cronbach’s α = 0.95).

**Table S3. Multiple Linear Regression Results Predicting Digital Addiction**

**Dependent variable: Digital addiction severity**

**N = 367**

| Predictor | B | SE | β | t | p | 95% CI  (Lower, Upper) |
| --- | --- | --- | --- | --- | --- | --- |
| Constant | 0.334 | 0.125 | — | 2.675 | .008 | [0.089, 0.580] |
| Age | 0.016 | 0.024 | 0.016 | 0.656 | .512 | [-0.031, 0.062] |
| Living arrangement | -0.006 | 0.024 | -0.006 | -0.254 | .800 | [-0.052, 0.040] |
| Daily use duration | 0.096 | 0.020 | 0.131 | 4.745 | <.001 | [0.056, 0.136] |
| X1  (Preferential incentives) | 0.190 | 0.036 | 0.227 | 5.273 | <.001 | [0.119, 0.260] |
| X2  (Interactive incentives) | 0.293 | 0.039 | 0.343 | 7.578 | <.001 | [0.217, 0.370] |
| X3  (Stage goals) | 0.142 | 0.044 | 0.160 | 3.200 | .001 | [0.055, 0.229] |
| X4  (Customized recommendations) | 0.149 | 0.039 | 0.163 | 3.850 | <.001 | [0.073, 0.225] |

**Model fit:** R² = 0.791, Adjusted R² = 0.787, F(7, 359) = 193.95, p < .001

**Note.** B = unstandardized coefficient; SE = standard error; β = standardized coefficient; CI = confidence interval.

**Table S4. Influence Statistics Summary**

| Statistic | Minimum | Maximum |
| --- | --- | --- |
| Cook’s distance | 0.000 | 0.112 |
| Centered leverage value | 0.002 | 0.134 |
| Studentized deleted residual | -5.565 | 2.601 |

No cases exceeded conventional thresholds for Cook’s distance (> 1) or leverage, indicating that no influential observations materially affected the regression results.

**Figure S1. Normal P–P Plot of Standardized Residuals
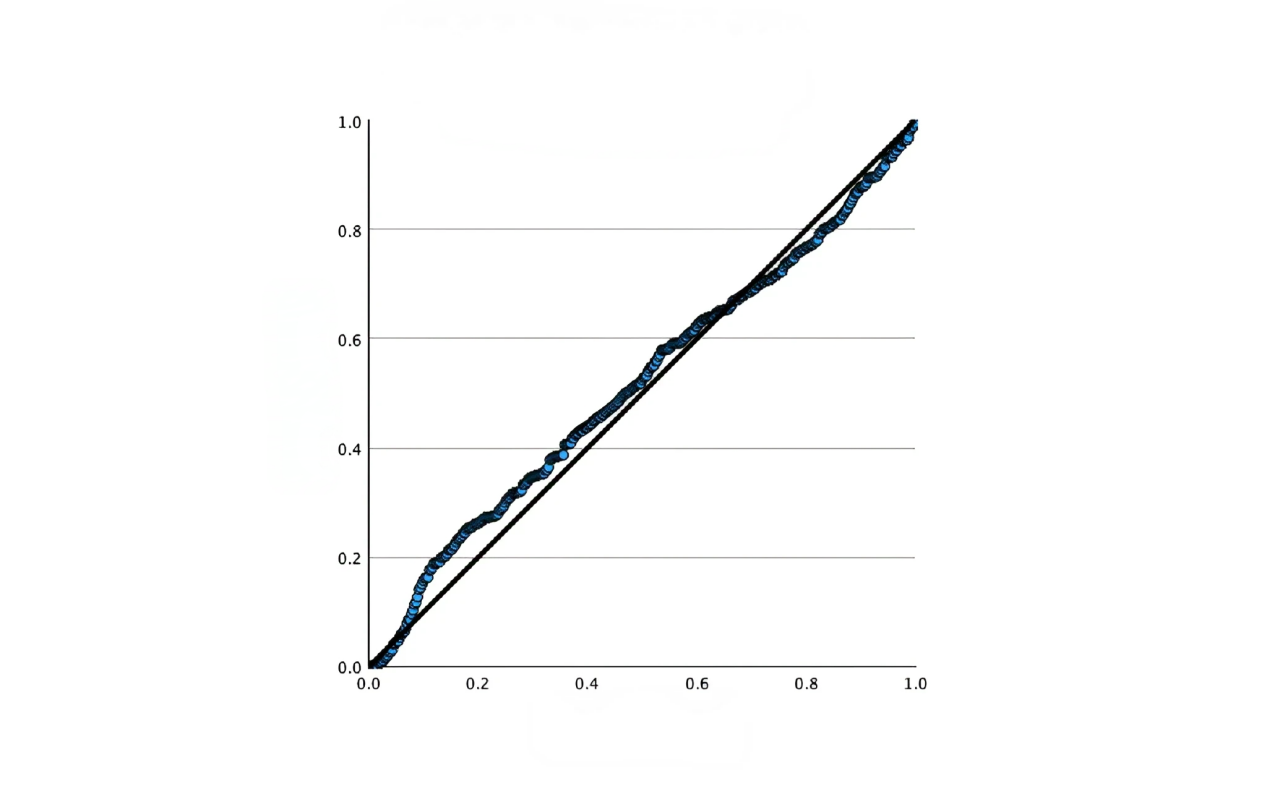
**

Caption：
The normal probability (P–P) plot of standardized residuals shows that the observed cumulative probabilities closely follow the diagonal reference line, indicating that the residuals are approximately normally distributed. This suggests that the normality assumption of the linear regression model is reasonably satisfied.

**Figure S2. Scatterplot of Standardized Residuals Versus Standardized Predicted Values**


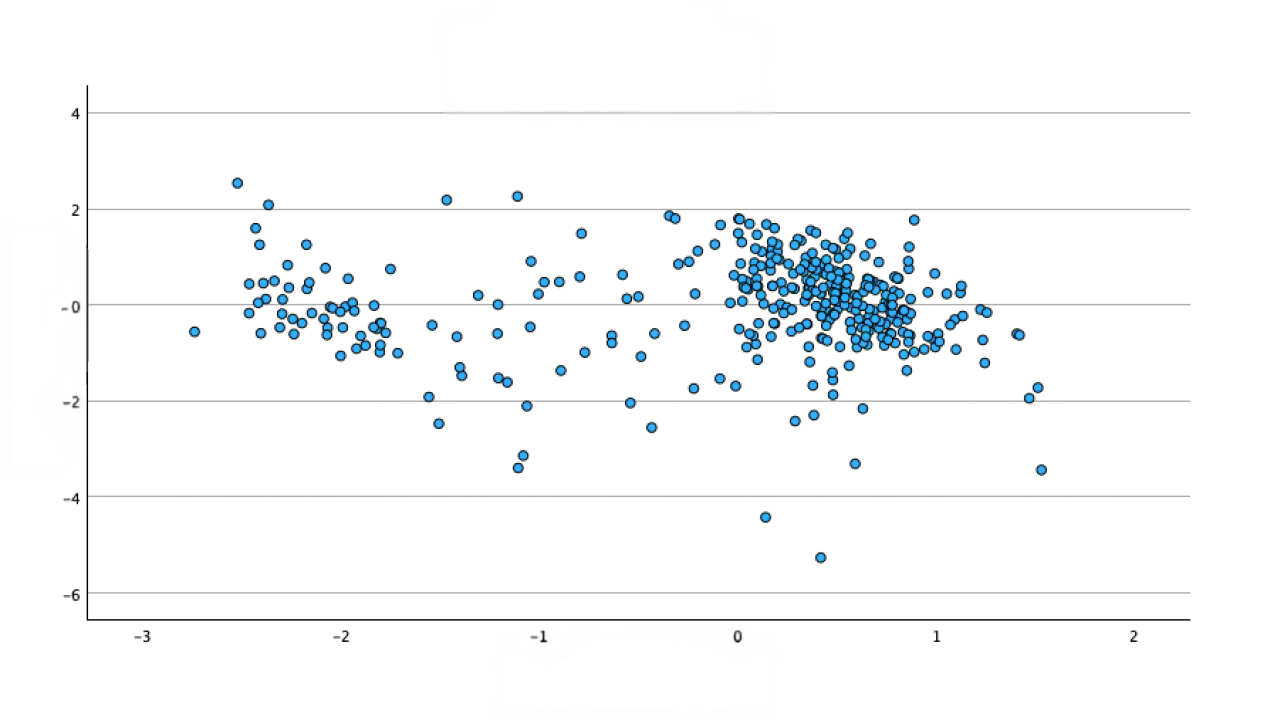
Caption：
The scatterplot of standardized residuals against standardized predicted values displays a random dispersion without a clear funnel-shaped or systematic pattern. This indicates no substantial heteroscedasticity and supports the assumption of homoscedastic residuals in the regression model. In addition, inspection of influence statistics (Cook’s distance and leverage values) did not identify any observations that would materially affect the regression results.
